# Unilateral Enlarged Vestibular Aqueduct Syndrome and Bilateral Endolymphatic Hydrops

**DOI:** 10.1155/2017/6195317

**Published:** 2017-05-18

**Authors:** Massimo Ralli, Giuseppe Nola, Luca Sparvoli, Giovanni Ralli

**Affiliations:** ^1^Department of Oral and Maxillofacial Sciences, Sapienza University of Rome, Rome, Italy; ^2^Operative Unit of ENT, GB Grassi Hospital, Ostia, Rome, Italy; ^3^Operative Unit of Radiology, Grassi Hospital, Ostia, Rome, Italy; ^4^Department of Sensory Organs, Sapienza University of Rome, Rome, Italy

## Abstract

Enlarged vestibular aqueduct (EVA) syndrome is a common congenital inner ear malformation characterized by a vestibular aqueduct with a diameter larger than 1.5 mm, mixed or sensorineural hearing loss that ranges from mild to profound, and vestibular disorders that may be present with a range from mild imbalance to episodic objective vertigo. In our study, we present the case of a patient with unilateral enlarged vestibular aqueduct and bilateral endolymphatic hydrops (EH). EH was confirmed through anamnestic history and audiological exams; EVA was diagnosed using high-resolution CT scans and MRI images. Therapy included intratympanic infusion of corticosteroids with a significant hearing improvement, more evident in the ear contralateral to EVA. Although most probably unrelated, EVA and EH may present with similar symptoms and therefore the diagnostic workup should always include the proper steps to perform a correct diagnosis. Association between progression of hearing loss and head trauma in patients with a diagnosis of EVA syndrome is still uncertain; however, these individuals should be advised to avoid activities that increase intracranial pressure to prevent further hearing deterioration. Intratympanic treatment with steroids is a safe and well-tolerated procedure that has demonstrated its efficacy in hearing, tinnitus, and vertigo control in EH.

## 1. Introduction

In adults, the vestibular aqueduct presents a diameter of 0.4–1.0 mm, with a mean value of 0.62 mm [1, 2]. Enlarged Vestibular Aqueduct (EVA), one of the most common congenital inner ear malformations, is characterized by a vestibular aqueduct with an anteroposterior diameter of 1.5 mm or more, measured halfway between the common crus and the operculum [3].

Clinical presentation includes audiological and vestibular symptoms that often mimic those of other middle and inner ear disorders such as otosclerosis [4, 5] and endolymphatic hydrops (EH) [6]. Mixed or sensorineural hearing loss (SNHL) is reported in 59–94% of cases, often associated with tinnitus and aural fullness. Hearing loss ranges from mild to profound, varying from fluctuating to progressive or sudden [7–11]; hearing fluctuations may happen following relatively minor head trauma. Mixed hearing loss may be supported by the hypothesis that an EVA introduces a third mobile window into the inner ear [12]. Vestibular symptoms in patients with an EVA syndrome have a prevalence between 14 and 73% depending on the study [7, 13–15] and range from severe episodic vertigo to occasional unsteadiness in adults, whereas incoordination and imbalance predominate in children [7, 16, 17].

Diagnosis of EVA syndrome is radiological. Computed Tomography (CT) scan shows the bony labyrinth anatomy, and an axial CT with 1.5-mm sections generally provides the best view of the vestibular aqueduct from the vestibule to the posterior surface of the petrous bone [8, 18]. Magnetic Resonance Imaging (MRI), especially on T2-weighted images, allows visualization of the membranous labyrinth [13, 14, 19] and is the only imaging technique that enables visualization of the extraosseous portion of the endolymphatic sac. Three-dimensional reconstructions from MRI data sets are often helpful in detecting the sac and other inner ear structures and to better define their morphological features, so that MRI is considered superior to CT in EVA evaluation by some authors [20, 21].

No treatment protocol for EVA syndrome was demonstrated to be uniformly successful in halting the progression of the disease; cochlear implantation is the optimal solution for hearing loss restoration when profound hearing loss is present [19].

Intratympanic corticosteroid treatment for inner ear diseases by direct injection in the middle ear has gained wide popularity in the last years [22–25], presenting several benefits such as an increased drug concentration in the target organ, reduced systemic steroid exposure, and reduced systemic adverse effects. The effects of inner ear corticosteroid therapy are based on their anti-inflammatory and immunosuppressive actions in addition to their regulatory role in ionic homeostasis as they act on potassium transport, improving the inner ear water balance [26].

Many audiovestibular symptoms found in EVA syndrome are in common with other inner ear disorders such as EH; differential diagnosis is therefore important for a correct diagnostic and therapeutic management of these patients. In this paper, we describe the case of a patient with a history of bilateral EH and a radiological diagnosis of EVA in the left ear, along with a detailed description of the diagnostic workup and therapeutic approach.

## 2. Case Presentation

A 39-year-old man was admitted to the ENT department of our institution with a four-year history of fluctuating bilateral SNHL, associated with acute objective vertigo, nausea, and vomit (4–8 episodes/year); the vertigo attacks, lasting from 15 minutes to three hours, were often accompanied by headache. The patient had no history of acoustic trauma and/or noise exposure and had a previous glycerol test positive for EH.

After admission, patient underwent a complete ENT examination with otoscopy, Pure Tone Audiometry (PTA), Acoustic Immittance Test, Transient Evoked Otoacoustic Emissions (TEOAEs), Distortion Products Otoacoustic Emissions (DPOAEs), Tympanometry, Cervical Vestibular Evoked Myogenic Potentials (cVEMPs), and caloric test.

PTA was carried out in a soundproof room and the pure tone thresholds for each side were measured at frequencies of 125, 250, 500, 750, 1000, 2000, 3000, 4000, 6000, and 8000 Hz; Air-Bone Gap (ABG) was measured at frequencies of 250, 500, 1000, 2000, and 4000 Hz. A standard 226 Hz tone tympanometry probe was performed to exclude external and middle ear pathologies. TEOAEs and DPOAEs were recorded in a sound attenuated chamber with an ILO-92 instrument (Amplifon, Milan, Italy). TEOAEs were evoked through 80–85 dB SPL stimuli, with a stimulation rate less than 60 stimuli per second, delivered through a probe inserted into the external auditory canal. DPOAEs were recorded with two acoustic stimuli (pure tones) at two frequencies (i.e., *f*1, *f*2 [*f*2 > *f*1]) and two intensity levels (i.e., *L*1, *L*2). cVEMPs were tested with the binaural simultaneous stimulation method, using an Amplaid MK22 polygraph (Amplifon, Milan, Italy). The electrodes were positioned as indicated by Colebatch et al. [27]; during the recording the patient was instructed to raise his head from the pillow to activate the bilateral sternocleidomastoid muscle. A stimulus at a frequency of 500 Hz was presented to one ear through a headphone at an intensity of 130 dB; the analysis window was 100 ms. Analysis was conducted on the amplitudes of the first positive–negative peak, P13–N23, and peak latencies of P13 and N23. The average of two measurements was taken to define amplitudes and latencies [28]. Caloric test was performed according to the Fitzgerald-Hallpike method: each ear was water-irrigated for 40 seconds at temperatures of 44°C and 30°C.

Diagnosis was completed through CT scan and MRI. CT scan was performed without contrast administration and using a helical acquisition technique: the temporal bone images were acquired with axial planes and evaluated on oblique, coronal, and sagittal planes. MRI images were obtained on a 1.5-T superconducting MR scanner (Philips INTERA). Targeted imaging of the vascular and nervous structures of the pontocerebellar angle were performed using axial 3-dimensional heavily T2-weighted images (3D TSE T2 WIs) and TSE T1 weighted images (TSE T1 WIs) with a slice thickness of 0.5 mm and 3 mm, respectively. Coronal T2 WIs were obtained using orthogonal planes to the long axis of the internal auditory canal and with oblique parasagittal and paracoronal planes (MPR reformatted images—slice thickness ranging between 0.4 mm and 3 mm).

**Figure 1:**
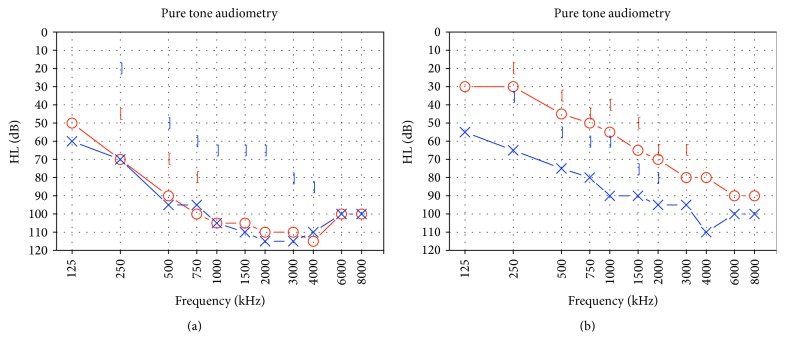
Pure tone audiometry results, before (a) and one month after (b) the intratympanic treatment. A bilateral hearing improvement, significantly more evident in the right side, is noticeable after treatment.

**Figure 2:**
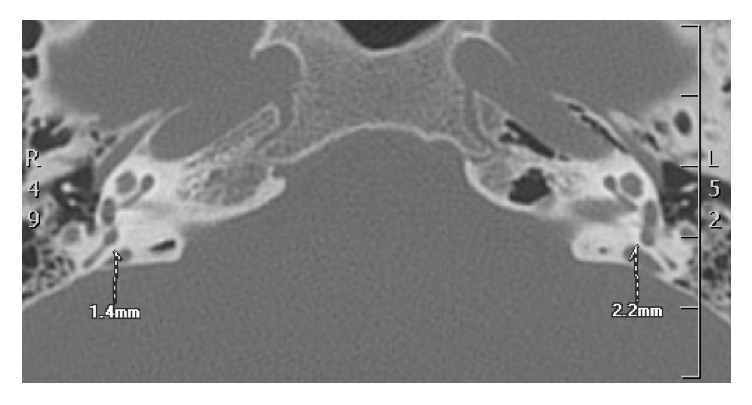
CT scan results: the endolymphatic duct and sac in the left side are larger (2.2 mm) than in the right side (1.4 mm).

**Figure 3:**
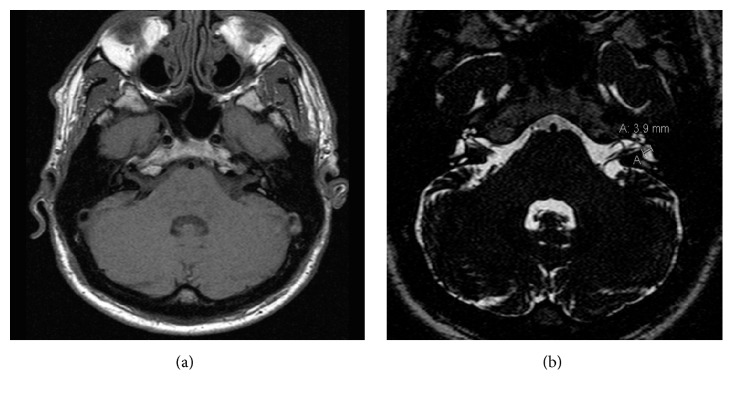
MRI. TSE T1 (a) and T2 WIs (b) images. The area of altered signal intensity in the left utricle is clearly defined while no abnormalities are seen in the right utricle.

PTA revealed a threshold of 95.9 dB and an ABG of 42.5 dB in the left ear and a threshold of 97.70 dB and an ABG of 17.5 dB in the right ear (Figure 1(a)). TEOAEs, DPOAEs, and VEMPs were absent bilaterally. Tympanometry presented a Type A tympanogram. The caloric labyrinth stimulation revealed bilateral normoreflexia. Audiological tests and history of bilateral fluctuating sensorineural hearing loss, more evident in the right side, and vertigo attacks were suggestive for a diagnosis of EH. Temporal bone CT revealed a 2.2 mm dilatation of the left vestibular aqueduct. A small (diameter: 2.6 mm) area of altered signal intensity was evident in the left vestibule (Figure 2). Enlarged endolymphatic ducts and sacs were seen on MRI (Figure 3) in the left side. CT and MRI images were also evaluated for cochlear dysplasia, cochlear-vestibular dysplasia, and modiolar hypoplasia based on published criteria [20, 29]. No additional inner ear malformations were observed in this patient.

Under local anesthesia (10% lidocaine, spray), patient was treated with bilateral intratympanic prednisone (5 mg/mL) once a day for three consecutive days, followed by 7 days of treatment suspension and additional 3 days of injections, using a 25-gauge spinal needle inserted in the posteroinferior portion of the tympanic membrane. Pure tone audiometry, TEAOEs, DPOAEs, tympanometry, VEMPs, and caloric test were repeated after 8 days and one, three, and six months. PTA values one month after the first injection were 86.8 dB with an ABG of 28.75 dB in the left ear and 62.7 dB with an ABG of 12.5 dB in the right ear (Figure 1(b)). Threshold did not significantly change at all follow-up time points. TEOAEs, DPOAEs, and VEMPs were bilaterally absent before and after intratympanic treatment. The caloric labyrinth stimulation revealed a bilateral normoreflexia at all time points.

## 3. Discussion

The causes of EVA syndrome are currently unknown. Different authors have hypothesized a blockade of inner ear development during the fifth week of embryonic life, when its growth is maximal, and an abnormal communication between the subarachnoid space and the inner ear [13].

Clinical manifestations in EVA syndrome are variable, suggesting that it may be related not only to anatomical abnormalities of the inner ear, but also to the physiology of the auditory and vestibular systems. In this case report, there are two important aspects to consider that, if missed, could lead to an incorrect diagnosis: the patient had a long-time history of bilateral hearing fluctuation and episodes of vertigo, suggestive for a diagnosis of bilateral EH. However, fluctuation in hearing can also be found in EVA patients, often following relatively minor head trauma; such fluctuations however are not usually associated with vertigo attacks [30]. In this patient, the long-time history of typical association of hearing fluctuation and vertigo crisis, associated with previously collected audiological evidence, including a positive glycerol test for EH and positive response to systemic and intratympanic therapy, can reasonably confirm the diagnosis of coexistent bilateral EH. The second element is the presence of bilateral hearing loss in a case of unilateral EVA. In such cases, hearing loss in the ear contralateral to the EVA is common; several authors reported that unilateral EVA may also present a contralateral hearing loss, suggesting that unilateral EVA may be a bilateral process despite unilateral imaging finding [30]. In this patient, however, while radiological evidence confirmed the diagnosis of left side EVA, audiological tests and, especially, history were also suggestive for a concomitant bilateral EH.

Although EVA is a congenital disorder, some authors proposed that hearing loss in EVA syndrome is acquired as it has been reported to be triggered by minor head trauma [31]. There is no agreement on the association between head trauma and hearing loss in EVA. Different authors suggested that cochlear injury could result from chemical damage to the organ of Corti by hyperosmolar endolymphatic sac content following reflux from the sac after head injury and by failure of the stria vascularis ion exchange mechanism [31, 32]. Another possible explanation could be found in a direct impact to the cochlea, causing a transient shockwave on the patient aqueduct followed by intracochlear membrane rupture, especially when abnormalities are present at this level [21]. A recent systematic review on progressive hearing loss and head trauma in EVA found that 39.6% of patients with SNHL in EVA syndrome report a history of head injury, and about 12% report a trauma-associated progression, concluding that although long-term progressive hearing loss is common in EVA syndrome, its association with head trauma is not strongly supported [31]. However, further histopathological studies are necessary for definitive conclusions.

The overall incidence of vestibular alterations in patients with EVA syndrome ranges from 12 to 86% [33]. Emmett reviewed 26 patients with EVA syndrome, reporting a 12% incidence of vestibular symptoms [7]; Jackler et al. [34] reported a 30% incidence of vestibular symptoms in a series of 17 patients; Berrettini et al. [35] found that 13/15 patients (86%) presented vestibular hypofunction or areflexia; Sugiura et al. [14] examined 17 patients with EVA syndrome, 12 of them (71%) referred with episodic vertigo. Vestibular dysfunction etiology is still unclear; it has been hypothesized that the reflux of hyperosmotic fluid into the basal end of the cochlear duct may elicit vertigo, while degeneration of vestibular hair cells due to osmotic and chemical imbalance may be another mechanism of injury [9, 28, 33]. Sheykholeslami et al. [36] measured VEMPs in three patients with an EVA syndrome who had previously undergone vestibular testing with normal results and demonstrated lower VEMP threshold scores in these patients, indicating a possible saccular dysfunction. In our study, VEMPs were bilaterally absent before and after intratympanic treatment therapy showing a permanent saccular damage.

In the comparison of MRI and CT scan for the diagnosis of an EVA syndrome, current literature suggests that both techniques are complementary for identifying structural alterations [31, 32]. MRI, however, presents some advantages: in fact, since the endolymphatic sac is not normally identifiable in patients without EVA, positive identification of this structure represents an easy diagnostic method. In addition, MRI provides a clear assessment of cochlear nerve integrity, central nervous system abnormalities, and the presence of nonossifying inner ear obstruction that is not evident on CT [18, 37]. MRI has also been proposed to diagnose EH. A study from Naganawa et al. showed that EH can be visualized using 3-T MRI performed 4 hours after intravenous injection of gadolinium [38]. Recently, Sone et al. investigated the presence of EH in subjects with EVA syndrome using 3T MRI and correlated imaging data concerning the degree of EH in the cochlea and the vestibule with clinical symptoms and hearing levels in 9 patients [39]. In this case, patient was studied using a 1.5T MRI that, due to its resolution, was unable to confirm EH; therefore, diagnosis was based on clinical and anamnestic data.

This patient was treated with intratympanic injection of corticosteroids with a partial hearing restoration, more evident in the right side, and an improvement in vertigo symptoms. As expected, the larger benefits in hearing restoration following corticosteroid treatment were seen in the ear contralateral to EVA. One of the first reports regarding the effects of intratympanic treatment of steroids for EH showed an 80% improvement in vertigo [39]. Afterwards, several studies on intratympanic treatment for EH have been published [23, 25, 40] showing different results on hearing and vertigo: the choice of steroids, the variability of their concentration, and the outcome measurements could explain the variability of the published results. Recently, Itoh and Sakata showed a significant control of vertigo in 82% of the treated patients after intratympanic treatment with dexamethasone (4 mg/ml, daily injection for 5 consecutive days) [41]. In the opinion of the authors, intratympanic injection for the treatment of labyrinthine affections such as EH is a procedure that maximizes drug concentration in the cochlea and minimizes systemic dissemination: the high concentration of topic steroids in the cochlea may justify the high percentage of remission observed in recent experiences.

In the literature, to the best of authors' knowledge, there is only one case report of a patient with EVA syndrome and EH, in which the authors hypothesized that the two conditions may be due to a common primary dysfunction of inner ear fluid homeostasis [42]. Although this physiopathological common basis cannot be confirmed, it is always necessary in patients with EVA to also investigate possible coexisting independent inner ear disorders such as EH, especially when a suggestive history for endolymphatic hydrops is present. Consistently, it is always necessary to perform a thoughtful radiological examination with CT scan and MRI in patients with audiovestibular symptoms suggesting an inner ear disorder.

Patients with a diagnosis of EVA, in the presence of serviceable hearing, should be advised to avoid contact sports or activities that increase intracranial pressure to prevent hearing loss or further hearing deterioration. Intratympanic treatment with steroids is a safe and well-tolerated procedure that has demonstrated its efficacy in hearing, tinnitus, and vertigo control in EH.
